# Metabolic syndrome and quality of life: a systematic review**^1^**

**DOI:** 10.1590/1518-8345.1573.2848

**Published:** 2016-11-28

**Authors:** Patrícia Pozas Saboya, Luiz Carlos Bodanese, Paulo Roberto Zimmermann, Andréia da Silva Gustavo, Caroline Melo Assumpção, Fernanda Londero

**Affiliations:** 2 PhD.; 3 PhD, Full Professor, Faculdade de Medicina, Pontifícia Universidade Católica do Rio Grande do Sul, Porto Alegre, RS, Brazil.; 4 PhD, Professor Adjunto, Faculdade de Enfermagem, Nutrição e Fisioterapia, Pontifícia Universidade Católica do Rio Grande do Sul, Porto Alegre, RS, Brazil.; 5 RN.

**Keywords:** Metabolic Syndrome X, Quality of Life, Risk Factors, Secondary Prevention, Cardiovascular Diseases, Metabolism.

## Abstract

**Objectives:**

to present currently available evidence to verify the association between metabolic syndrome and quality of life.

**Method:**

Cochrane Library, EMBASE, Medline and LILACS databases were studied for all studies investigating the association with metabolic syndrome and quality of life. Two blinded reviewers extracted data and one more was chosen in case of doubt.

**Results:**

a total of 30 studies were included, considering inclusion and exclusion criteria, which involved 62.063 patients. Almost all studies suggested that metabolic syndrome is significantly associated with impaired quality of life. Some, however, found association only in women, or only if associated with depression or Body Mass Index. Merely one study did not find association after adjusted for confounding factors.

**Conclusion:**

although there are a few studies available about the relationship between metabolic syndrome and quality of life, a growing body of evidence has shown significant association between metabolic syndrome and the worsening of quality of life. However, it is necessary to carry out further longitudinal studies to confirm this association and verify whether this relationship is linear, or only an association factor.

## Introduction

Metabolic Syndrome (MS), understood as a complex set of cardiovascular risk factors, related to abdominal fat accumulation and resistance to insulin, is strongly associated with high cardiovascular morbimortality^1^ ^-^ ^4^, even when type 2 diabetes is not present^4^. The analysis of the Heart Outcomes Protection Evaluation - HOPE study corroborated this idea and adds that the increase in the risk is directly and progressively associated with the increase in waist circumference (WC)^5^.

Several definitions of MS have come up along these years, although they show some variations concerning criteria and reference values for the metabolic parameters connected with the syndrome. According to The Third Report of The National Cholesterol Education Program (NCEP-ATP III), definition recommended by I Brazilian guidelines on diagnosis and treatment of MS, the presence of alterations in 3 out of 5 risk factors, such as abdominal obesity (AO); WC >102cm for men and >88cm for women, triglycerides ≥150mg/dl; HDL cholesterol <40mg/dl for men and <50mg/dl for women, Blood Pressure ≥130/85 mmHg and fasting glucose ≥110mg/dl, would form the MS diagnosis, regardless of the presence of glycemia^1^ ^-^ ^2^.

Nevertheless, considering the available evidence on the connection between central obesity and risk for cardiovascular disease, the International Diabetes Federation (IDF) published in 2005 a new MS criterion, requiring the presence of AO as well as 2 or more criteria for the MS diagnosis, also proposing reduction in the WC reference values ≥94cm for men and ≥80cm for women, and glucose levels ≥100 mg/dl^3^.

The incidence level of MS has been increasing progressively in the last decades, estimating a prevalence of up to 23.7%, according to ATP III criteria when adjusted for age, according to a study carried out in the USA with a sample of 8.814 adults^6^.

Despite all progress made in understanding and treating MS, it is still an important public health issue. Moreover, the study of the impact of MS on the quality of life (QOL) has been receiving little attention in medical literature and because of that, is still controversial and is not well understood. Our aim was to present currently available evidence for all studies investigating the effects of the MS on the QOL to verify the association between MS and the QOL.

## Method

The databases searched were the Cochrane Library, EMBASE, Medline and LILACS through 1988 to present, using the following key words: Metabolic Syndrome X, Risk Factors and Quality of Life, for all studies investigating the effects of the MS on the QOL. References from the above studies not identified in the database search were also surveyed.

The study selection considered: adults of both sexes and all studies published in English, Spanish and Portuguese language. Those who had a small sample size or presented another important associated disease were excluded to avoid possible biases. Studies not meeting these criteria were excluded.

The data were extracted by two blinded reviewers and were subjected to qualitative analysis. Disagreements were resolved by consensus, but one more reviewer was chosen in case of doubt. Reviewers extracted information on authors, publication year, sample size, study design, including the duration of follow-up, and results.

**Figure 1:**
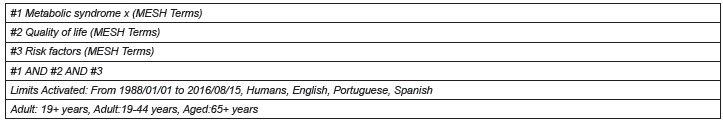
Search strategy in the Medline/ Pubmed databases

The search strategy adopted in the Medline, which was also used for the other databases analyzed, is presented in the Figure 1.

## Results

Although there are few studies in this area, most of them show association between MS and worsening in QOL^7^ ^-^ ^16^, even more significant when regarding subjects who also have depression^17^. 

However, a cross-sectional study assessing 390 obese patients, out of which 269 filled MS criteria, showed that MS in itself was not associated with a reduction in QOL, but only showed significant correlation when associated with other factors, such as depression^18^.

A recent study involving 4.480 subjects revealed that the number of components diagnosed with MS was inversely associated with General Health, in both genders, although it was positively associated with Mental Health^7^. Another two studies^19^ ^-^ ^20^ corroborated the idea of the impact of the MS components on the worsening of QOL, more specifically in the domains of Physical Health, although the studies demonstrated that this association is only significant in women.

Similarly, cross-sectional studies reveal that this association between MS and QOL differs according to the gender^19^ ^-^ ^24^.

According to the results of a cross-sectional study with 4.463 subjects of both genders, the decrease in QOL scores is directly proportional to the increase in the number of MS components in men as well as women, although this association is significant only in women^21^.

Likewise, two other cross-sectional studies with 950 and 2.264 subjects of both genders, respectively, also showed that this association between MS and decrease in the QOL scores was only significant in women^20^ ^,^ ^22^. 

Results of a Swedish study with 1.007 men and women with MS, although showing lower scores in the physical and social domains of the Medical Outcomes Study Short Form, General Health Survey (SF-36) in subjects of both genders, showed that, after adjustments for confounding factors, such as age, smoking, physical activity, etc., this difference was also significant in women. This study also revealed that there were no differences for mental health or perceived stress between subjects with and without MS^23^. 

Similarly, even though another instrument for measuring QOL was used, a cross-sectional study with 9.570 men and women from Iran also showed association between QOL domains (social relation and physical health) and MS only in women, after adjusted for confounding factors^24^.

In addition, cross-sectional studies carried out with women^25^, with significant samples of 6.913^26^ and 6.805^27^ subjects respectively showed significant association between MS and worsening of QOL. Although some studies show this association, it only takes place in the Physical Health domain of QOL^26^ ^-^ ^27^.

Recent studies of intervention in order to change MS patients' lifestyles already show results of significant improvement in QOL^28^ ^-^ ^33^. 

A randomized controlled trial with 201 obese women with 1 or more MS components, followed by 12-month, demonstrated that, after the intervention, the prevalence of MS decreased and the QOL scores increased in most domains in the group of intensive intervention, compared to the group of moderate intervention^29^. 

 According to data from another randomized controlled trial with 390 obese patients of both genders that showed at least two MS criteria, after the 6th month of intervention there was significant improvement in several QOL domains of the SF-36, and this association was more significant in women in the 24-month follow-up^28^.

Similarly, an intervention study conducted in Brazil with a 9-month follow-up also showed significant improvement in QOL scores in most SF-36 domains, especially in the group of intensive intervention^30^. 

Another two randomized controlled trials followed by 1 year, also demonstrated significant improvement in QOL, specially in Mental Health domains^31^ ^-^ ^32^.

However, a 12-week Hatha yoga intervention on MS showed not only beneficial changes in Mental Health (social functioning), but also in Physical Health (general health and Physical component score)^33^.

A cohort of 1.785 subjects, showed that low QOL scores, in the physical health domain of the SF-36, were associated with MS and significantly predicted 5-year mortality^34^. 

Similarly, another cohort study with 657 subjects, during 7 years, also showed that MS, anxiety and depressive symptoms are independent predictors of poorer subjective health and QOL. MS was associated with weaker self-rated health in men, but weaker perceived life satisfaction in women^35^.

However, a cross-sectional analysis of a study with 361 subjects in two weight-loss programs revealed that, although an association between MS and low scores of QOL were found only in the physical health domain of the SF-36, this association was not kept after being adjusted for BMI, which means that this QOL worsening will be explained by the BMI increase and not by the MS itself^36^. 

Nevertheless, only one cross-sectional study did not find significant association between MS and QOL after adjustments such as age, gender, smoking and so forth^37^.

**Figure 2:**
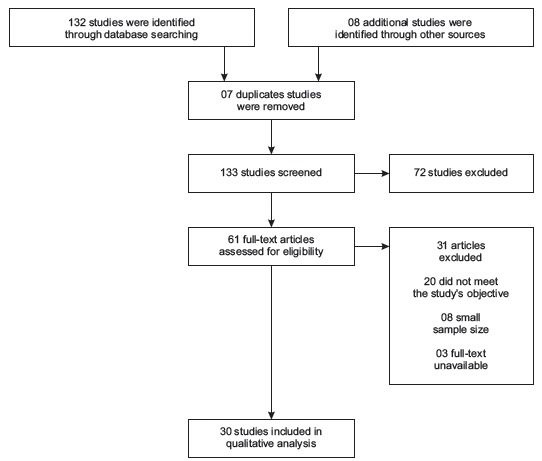
Flow chart of selection studies

A total of 133 studies were screened, however only 61 were assessed for eligibility and only 30 were included in this review, which included 62.063 patients (Figure 2).

**Figure 3:**
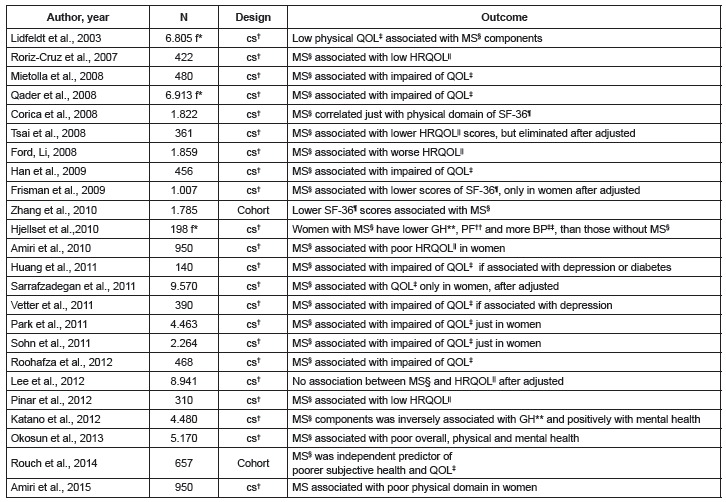
Observational studies reporting the relation between metabolic syndrome and quality of life

The Figures 3 and ^4^show observational and clinical trials studies, respectively reporting the association between MS and quality of life.

* f = Female, † cs = Cross-sectional, ‡ QOL = Quality of life, § MS = Metabolic syndrome, || HRQOL = Health-Related Quality of Life, ¶ SF-36 = Medical Outcome Study Short Form, General Health Survey (SF-36), ** GH = General Health, †† PF = Physical Functioning, ‡‡ BP = Bodily Pain

**Figure 4:**
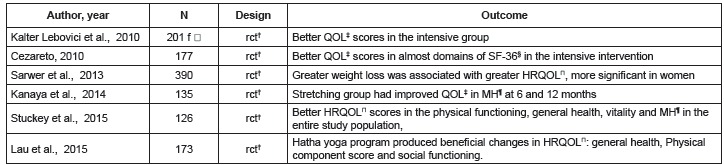
Clinical trials reporting the relation between metabolic syndrome and quality of life

* f = Female, † rct = Randomized controlled trial, ‡ QOL = Quality of life, § SF-36 = Medical Outcome Study Short Form, General Health Survey (SF-36), | HRQOL = Health-Related Quality of Life, ¶ MH = Mental health

Among the instruments used for measuring health-related quality of life (HRQOL), the SF-36 was the most frequently used comprising a total of 16 out of the 30 selected studies, though 3 of them have been used in its reduced version.

**Figure 5:**
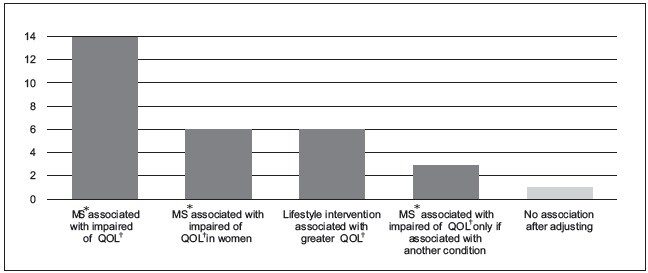
All studies reporting Metabolic syndrome and quality of life

Almost all studies suggested that MS is significantly associated with QOL. Some, however, found association only in women, or only if associated with depression or BMI. Merely one study did not find association after adjusted for confounding factors. Graphical representations of results are shown in Figure 5.

* MS = Metabolic syndrome, † QOL = Quality of life

## Discussion

Our systematic review was designed to provide an overview of what is known about the association between MS and QOL.

Unfortunately, there is still little evidence as well as some problems concerning a high proportion of cross-sectional studies and different HRQOL instruments have contributed to the lack of evidence. 

In addition, it was observed in this review, different study populations. Once these studies come from different countries with several cultures and lifestyles, it is difficult to generalize the data found.

Nevertheless, a growing body of evidence demonstrates significant association between MS and worsening in the QOL, more specifically in women. It is necessary to carry out further longitudinal research to determine if this relationship is linear, or only an association factor. 

Another important factor that needs to be investigated refers to a more precise identification of the QOL domains that are more affected by the presence of MS. Few studies refer to these data, once different instruments are used to measure this variable.

On the other hand, recent intervention studies already show improvement in the metabolic parameters and quality of life based on programs for changing lifestyles, which may contribute to a future intervention strategy. However, there is still doubt whether these findings remain after the intervention. 

All things considered, we note that the study of the relationship between MS and QOL, due to its relevance been receiving little attention in medical literature.

The present review has some limitations: the design of the studies, i.e., a high proportion of cross-sectional studies and the different HRQOL instruments used.

## Conclusion

Although there are a few studies available about the relationship between MS and QOL, a growing body of evidence has shown significant association between metabolic syndrome and the worsening of quality of life. Similarly, lifestyle interventions in individuals with MS demonstrated improvement of MS and better QOL scores.

However, it is necessary to carry out further longitudinal studies to confirm this association and verify whether this relationship is linear, or only an association factor.

The contribution of the present study was to draw attention to the effects that the MS can have on QOL, in an attempt to improve prevention and treatment strategies for MS, considering the fact that MS is still an important public health issue.
